# Mucosal color changes on narrow-band imaging in esophageal eosinophilic infiltration

**DOI:** 10.1097/MD.0000000000029891

**Published:** 2022-09-23

**Authors:** Tsuyoshi Suda, Yukihiro Shirota, Yuji Hodo, Katsuaki Sato, Tokio Wakabayashi

**Affiliations:** a Department of Gastroenterology, Saiseikai Kanazawa Hospital, Ishikawa, Japan; b Department of Pathology II, Kanazawa Medical University, Ishikawa, Japan.

**Keywords:** eosinophilic esophagitis, gastroesophageal reflux, mucous membranes, narrow-band imaging, retrospective studies

## Abstract

This study aimed to examine the range of beige colored mucosa (BCM) in patients with esophageal eosinophilic infiltration (EEI) using narrow-band imaging (NBI).

In this retrospective study, EEI was confirmed histologically in 12 consecutive patients from January 2014 to December 2017. The BCM tone on NBI without magnifying endoscopy was evaluated, and red, green, and blue (RGB) values of BCM and normal mucosa were measured. BCM was macroscopically classified into 2 groups (bright and dark) using cluster analysis. Histopathological analysis was performed in 1 patient who underwent biopsy for both normal mucosa and BCM.

All 12 patients presented with BCM. Endoscopy revealed fixed rings, longitudinal furrows, mucosal edema, and exudate in 3, 12, 10, and 8 patients, respectively. Strictures were absent. Five patients had findings suggestive of gastroesophageal reflux disease. In the cluster analysis, 5 and 7 patients had bright and dark BCM, respectively. Consistent results were noted when we categorized patients according to their macroscopic characteristics. RGB values of the BCM and normal mucosa were measured—normal mucosa: R: 99.8 ± 16.5, G: 121.7 ± 23.1, and B: 93.4 ± 19.2; BCM: R: 152.0 ± 31.3, G: 123.9 ± 35.0, and B: 97.5 ± 29.5. BCM had significantly higher R values than normal mucosa (*P* = .0001). All parameters were significantly lower in the dark BCM group than in the bright BCM group (*P* < .001). Histopathological analysis revealed expansion of the epithelial intercellular space, eosinophilic infiltration, and basal cell hyperplasia at the BCM sites.

BCM was observed in all cases of EEI. RGB values differed between bright and dark BCM. Assessing BCM tone using NBI is a potentially novel diagnostic method for EEI.

## 1. Introduction

Eosinophilic esophagitis (EoE) is a rare allergic disorder characterized by esophageal motor dysfunction and organic stenosis. This condition is caused by eosinophil infiltration of the esophageal epithelium.^[1]^ Endoscopic findings, such as linear furrows, concentric rings, or white exudates, have been reported in cases of EoE.^[1,2]^

A previous study reported that narrow-band imaging (NBI) without magnifying endoscopy (ME) does not improve the efficacy of diagnosing EoE in terms of detecting endoscopic findings.^[3]^ However, other studies have reported specific findings in EoE using NBI-ME. In these reports, increased area of the beige colored mucosa (BCM) (normal mucosa has a light green color), dot-shaped congested intraepithelial papillary capillary loops (IPCLs), and invisibility of submucosal vessels (normal mucosa has cyan-colored vessels) were described as characteristic findings.^[4,5]^ However, further research on the use of NBI in diagnosing EoE has not been reported.

Furthermore, some reports have suggested that mucosal injuries due to gastroesophageal reflux disease (GERD) are related to the risk of EoE. Other reports have also suggested that localized or early-stage EoE could not be distinguished from GERD clinically or histopathologically.^[6,7]^ Therefore, esophageal eosinophilic infiltration (EEI) related to GERD is also becoming clinically relevant. We found a wide range of BCM tone in EEI using NBI, and this phenomenon was recognized clearly without NBI-ME. Therefore, the aim of this study was to examine this phenomenon.

## 2. Methods

### 2.1. Study design and patients

This retrospective study was conducted at a single center (Saiseikai Kanazawa Hospital, Japan). Twelve consecutive patients who were diagnosed with EEI after undergoing esophageal biopsies between January 2014 and December 2017 were included in this study. EEI was confirmed histologically by the presence of ≥15 eosinophils/high-power field (HPF) in at least 1 esophageal biopsy sample. If most eosinophils were degranulated, a pathologist was asked to confirm the diagnosis. The study protocol was reviewed and approved by the Institutional Review Board (IRB) of the participating institution. The requirement for obtaining informed consent from the patients was waived by the IRB due to the retrospective design.

Endoscopic images of all patients were reviewed, and the tone of BCM using NBI without ME was evaluated by an endoscopist. BCM was macroscopically classified into the following 2 groups: bright BCM and dark BCM. Bright BCM was defined as a visibly bright brown color, whereas dark BCM was defined as a visibly dark brown.

### 2.2. Endoscopic system and setting

The endoscopic system and devices included were as follows: an upper gastrointestinal endoscope (GIFH290, Olympus Medical Systems, Tokyo, Japan), a video processor (EVIS LUCERA Olympus CV-260SL, Olympus Medical Systems), and a light source (EVIS LUCERA Olympus CLV-260SL, Olympus Medical Systems). The structure enhancement of the endoscopic video processor was set to B-mode level 8.

### 2.3. Histopathological investigation

A pathologist evaluated each esophageal biopsy sample. Furthermore, the pathologist evaluated the presence or absence of an association between epithelial intercellular space expansion, eosinophil infiltration, basal cell hyperplasia, red blood cell (RBC) leakage, and BCM.

### 2.4. Red, green, and blue values

Red, green, and blue (RGB) values on the endoscopic images were measured using a free software (Paint 3D, Microsoft Corporation, San Diego, CA). The most characteristic tone of BCM was measured on both light and dark BCM. For example, the points of the lightest and darkest brown were measured for bright and dark BCM, respectively. The measured point for normal mucosa on NBI was selected based on observing normal capillaries.

### 2.5. Statistical analysis

RGB values were compared using Student *t*-test (*t*). *P* < .05 was considered statistically significant. All analyses were performed using the GraphPad Prism ver. 7.00 for Windows (GraphPad Software, San Diego, CA).

K-means clustering was used to divide the color of BCM into 2 groups using statistical software (College Analysis Ver. 7.8, https://www.heisei-u.ac.jp/ba/fukui/analysis.html, Japan). This analysis is one of the simplest and most common unsupervised clustering algorithms. Data mining began with the first randomly selected group centroid; these centroids were used as the starting point for all clusters. The calculation for optimizing the position of the center of gravity was subsequently repeated. The analysis was terminated when the center of gravity was stable or the defined number of iterations was achieved.

## 3. Results

Twelve patients (10 men and 2 women) with a mean age of 49 (range, 35–59) years were included in this study. Five patients (42%) met the updated international consensus diagnostic criteria for EoE.^[8]^

Regarding clinical symptoms, 2 patients had food impaction, and 3 experienced heartburn (Table 1). Endoscopic findings revealed fixed rings in 3 patients, longitudinal furrows in 12 patients, mucosal edema in 10 patients, and exudate in 8 patients. Strictures were absent, and 5 patients (42%) had findings suggestive of GERD.

**Table 1:** Patients’ characteristics.

Sex	n, (% or range)
Male	10 (83)
Female	2 (17)
Mean age	49 (35–59)
Symptoms	
Dysphagia	0 (0)
Food impaction	2 (17)
Heart burden	3 (25)
Endoscopic findings	
Fixed rings	3 (25)
Longitudinal furrows	12 (100)
Stricture	0 (0)
Mucosal edema	10 (83)
Exudates	8 (67)
GERD	5 (42)
EoE diagnosis	5 (42)
NBI observations	
Appearance of BCM	12 (100)
“bright”*	5 (42)
“dark”*	7 (58)

BCM = beige color of the mucosa, EoE = eosinophilic esophagitis, NBI = narrow-band imaging, GERD = gastroesophageal reflux disease.

* K-means clustering.

Features suggestive of BCM were observed in all patients. The RGB values of BCM and normal mucosa were measured (Table 2). The RGB values obtained for the normal mucosa were R: 99.8 ± 16.5, G: 121.7 ± 23.1, and B: 93.4 ± 19.2, and those obtained for the BCM were R: 152.0 ± 31.3, G: 123.9 ± 35.0, and B: 97.5 ± 29.5. The *R* value of the BCM was significantly higher than that of the normal mucosa (*P* = .0001, Student *t*) (Table 2, see Figure, Supplemental Digital Content 1, http://links.lww.com/MD/G897).

**Table 2:** The RGB value of BCM and normal mucosa.

	R	G	B
BMC	152.0 ± 31.3	123.9 ± 35.0	97.5 ± 29.5
“Bright”	173.6 ± 12.0*	152.6 ± 10.7‡	121.6 ± 9.2‡
“Dark”	127.0 ± 19.3†	93.3 ± 12.5	72.3 ± 10.7
Normal	99.8 ± 16.5	121.7 ± 23.1	93.4 ± 19.2

Mean ± SD.

B = blue, BCM = beige color of the mucosa, G = green, R = red.

* *P* = .0001.

† *P* = .0004.

‡ *P* < .0001: Student *t*-test.

Furthermore, the tone of the BCM showed a varying distribution across cases. We noticed that there were bright and dark BCM macroscopically. Thus, we classified the RGB values of BCM into 2 groups using the k-means clustering method. The result of this analysis categorized all 12 patients into 2 groups, with 5 and 7 patients with bright and dark BCM, respectively. Surprisingly, this result was consistent with our finding when patients were classified according to their macroscopic characteristics (Table 1).

Comparing the bright and dark BCM, the RGB values of the bright BCM were R: 173.6 ± 12.0, G: 152.6 ± 10.7, and B: 121.6 ± 9.2, whereas those of the dark BCM were R: 127.0 ± 19.3, G: 93.3 ± 12.5, and B: 72.3 ± 10.7 (Table 2). All parameters were significantly lower in the dark BCM group than in the bright BCM group (*P* < .001, Student *t*) (Table 2, see Figure, Supplemental Digital Content 2, http://links.lww.com/MD/G897).

**Figure 1.**
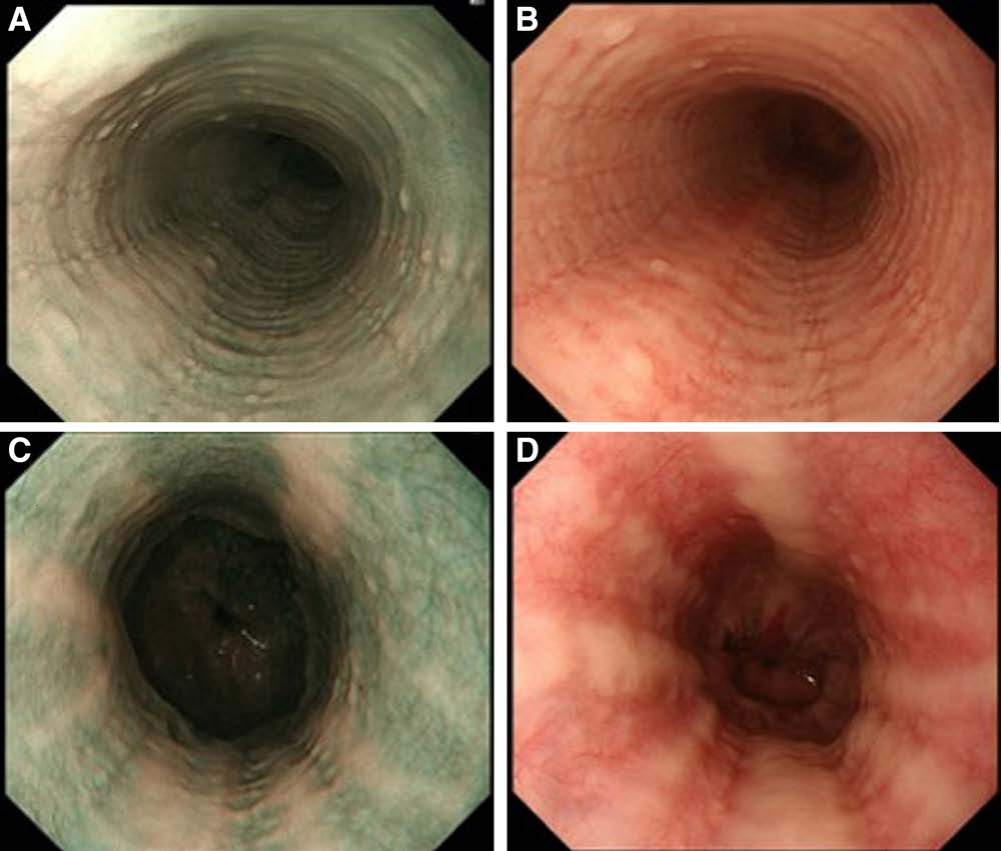
“Bright” beige colored mucosa (BCM). Case 1: Eosinophilic esophagitis (EoE) was confirmed via biopsy. A. Bright BCM revealed using narrow-band imaging (NBI). B. BCM appears to correspond to mucosal edema under white light; however, the border is unclear. Case 2. Esophageal eosinophilic infiltration (EEI) was diagnosed using biopsy; however, EoE was not diagnosed clinically due to the absence of symptoms. C. Bright BCM observed radially using NBI. D. Mucosal edema is confirmed under white light, unlike in case 1.

**Figure 2.**
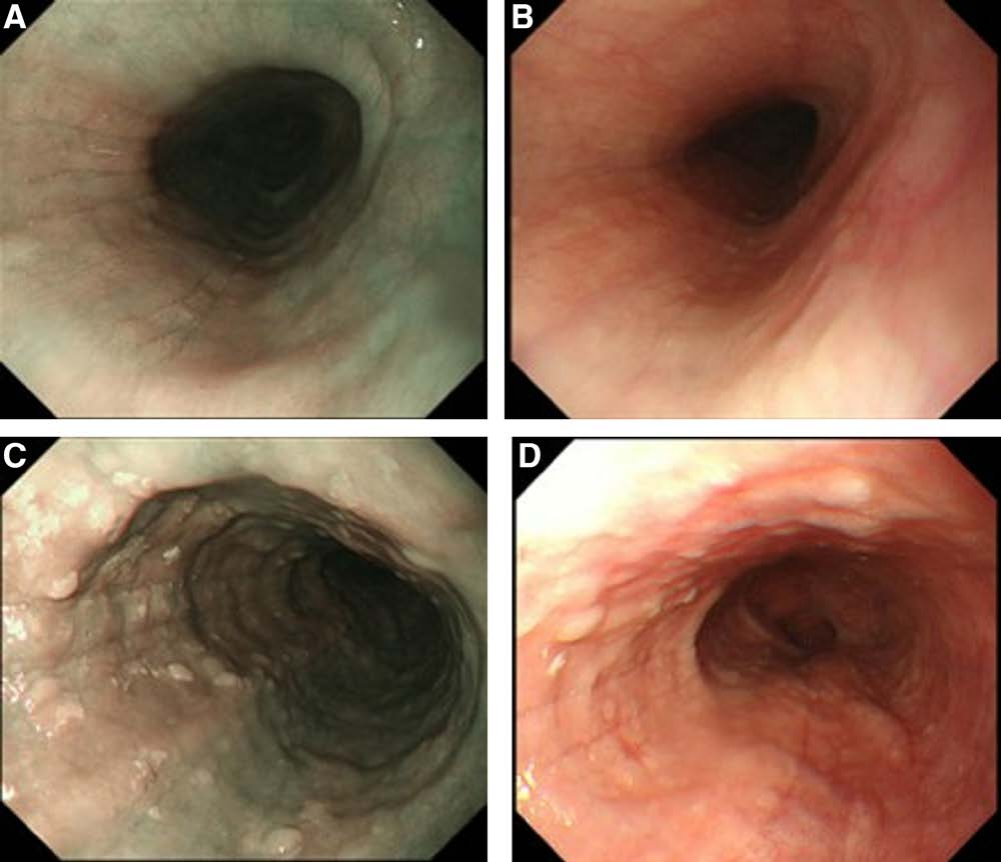
“Dark” beige colored mucosa (BCM). Case 3: Eosinophilic esophagitis (EoE) was diagnosed via biopsy. A. The border is unclear, as in case 1. B. EoE was suspected following biopsies and histopathological examinations. Dark BCM is noted on the left side of the esophageal mucosal wall using narrow-band imaging (NBI). Case 4: Esophageal eosinophilic infiltration (EEI) was diagnosed using biopsy; however, EoE was not diagnosed clinically due to the absence of symptoms. C. Dark BCM is located on the left esophageal wall on NBI. D. Mucosal edema is confirmed using white light.

The following cases are the representative samples: cases 1 and 2 included bright BCM. In case 1, a diagnosis of EoE was established (Fig. 1A). Under white light, the BCM appeared similar to mucosal edema; however, the border was not clear (Fig. 1B). In case 2, EEI was diagnosed but not EoE, due to the absence of symptoms. BCM was confirmed to be mucosal edema under white light, unlike in case 1 (Fig. 1C, D). Cases 3 and 4 included dark BCM. In case 3, EoE was diagnosed, and the border of the BCM was not clear (Fig. 2A, B). In case 4, EEI was diagnosed but not EoE, due to the absence of symptoms. Dark BCM was noted on the left wall of the esophageal mucosa on NBI (Fig. 2C), and it appeared similar to mucosal edema under white light (Fig. 2D).

**Figure 3.**
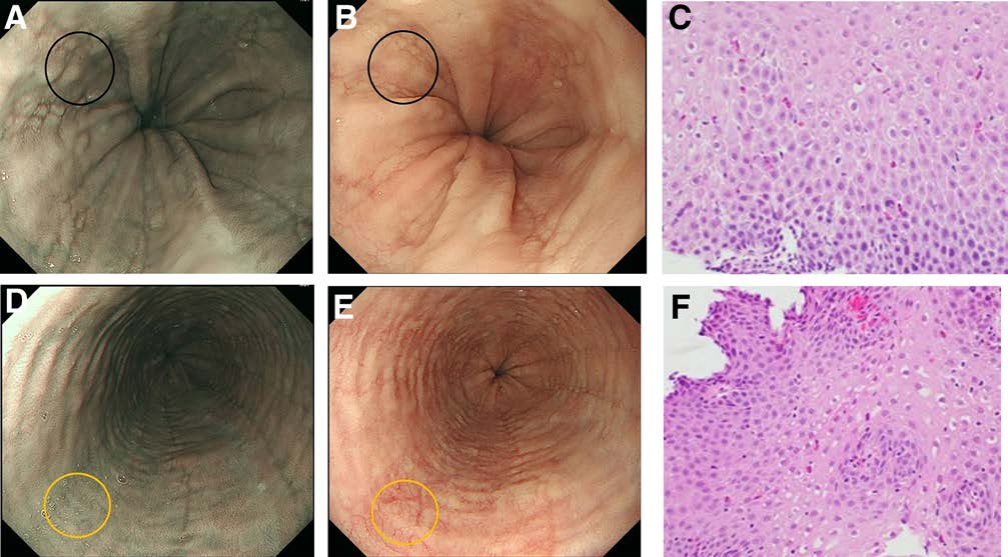
Pathological analysis. A. Beige colored mucosa (BCM) was observed using narrow-band imaging (NBI). B. The area of BCM is recognized as mucosal edema under white light. C. Expansion of the epithelial intercellular space, eosinophil infiltration, and basal cell layer hyperplasia are pathologically observed. D–F. The normal mucosa between the areas with BCM shows no expansion of the epithelial intercellular space; however, slight eosinophilic infiltration is observed.

Histopathological analysis was also performed in 1 patient who underwent biopsy of the normal mucosa, and BCM was discovered accidentally. The site of the BCM on NBI (Fig. 3A) was identified as mucosal edema under white light (Fig. 3B), as it demonstrated epithelial intercellular space expansion, eosinophil infiltration, and basal cell hyperplasia (Fig. 3C).

The normal mucosa between areas with BCM (Fig. 3D, E) had no expansion of epithelial intercellular spaces but had mild infiltration of eosinophils (Fig. 3F) on histopathological examination. These findings suggested that BCM reflected an expansion of the epithelial intercellular space. Unfortunately, leakage of RBCs was not confirmed in this specimen.

## 4. Discussion

The prevalence of EoE is approximately 30/100,000 (0.03%) in Europe and America^[9]^ and 17.1/100,000 (0.017%) in Japan.^[10]^ However, its prevalence is thought to have increased rapidly in recent years.^[11]^ Several asymptomatic patients have demonstrated characteristic EoE findings during esophagogastroduodenoscopy (EGD). A Swedish study reported that the prevalence of EoE (>15 eosinophils/HPF) was 11 in 1000 patients (1.1%) in the general population who underwent EGD; however, approximately half of them were asymptomatic.^[12]^ Furthermore, 25 to 40% of patients with EoE in Japan are asymptomatic.^[13–15]^ Although fixed rings, longitudinal furrows, stricture, mucosal edema, and exudates are characteristic endoscopic findings of EoE,^[1,2]^ detecting the condition in asymptomatic patients is difficult if the endoscopist does not recognize the characteristic features.

When EoE is diagnosed, it is important to confirm the diagnosis through biopsy. EoE is defined by the American Gastroenterological Association as the presence of > 15 eosinophils/HPF.^[8]^ However, a diagnosis of EoE requires other findings to differentiate it from other diagnoses. Particularly, symptoms of EoE are confused with those of GERD, such as heartburn. EoE is caused by acidophilic inflammation because of esophageal mucosal injury and increased permeability due to allergies, unlike GERD.^[16]^ However, gastric acid reflux also causes mucosal injury, thus suggesting that mucosal injury plays an important role in the development of EoE.^[5]^ Differentiating localized, early-stage EoE from GERD is difficult because of overlapping clinical and histopathological features.^[6]^ This supports the finding showing that some patients with EoE respond to proton pump inhibitor (PPI) therapy.^[17]^ Cases of EEI according to GERD are expected to increase; therefore, it is important to effectively determine the characteristics of EEI.

Although BCM was reportedly first detected using NBI-ME, it can also be recognized using NBI without ME. The representative samples included in this study demonstrated that BCM, when examined using NBI, appeared as regions of mucosal hyperplasia with mucosal edema and decreased vascular permeability, thus indicating that NBI cannot adequately diagnose EoE.

However, the standard method of identifying the tones of BCM remains unknown. In contrast to the traditional imaging methods, NBI is an image enhancement technology that dramatically improves the contrast visibility of capillaries on the surface of mucous membranes by exposing the mucous membranes to 2 different visible narrow-band wavelengths (center wavelengths: 415 and 540 nm). Enhancing the contrast between the capillaries on the surface of the mucous membrane and the background mucosa is achieved using light wavelength bandwidths that are well absorbed by hemoglobin (Hb) in the blood and have strong light-scattering properties. Given that the light with a shorter wavelength within the visible spectrum has stronger light-scattering properties than the light with a longer wavelength, the former’s penetration into the submucosa is low. Additionally, light absorption by Hb in the blood is maximized close to 415 nm. Therefore, 415 nm light is strongly absorbed by the capillaries that run through the shallow mucous membrane tissues; thus, resulting in improved visibility of the capillaries against the background mucosa. The 540 nm light is used to differentiate between the shallow capillaries and the thicker blood vessels located deeper in the mucous membrane.^[18]^ In terms of pathology, although EEI has characteristic features, especially a high permeation of eosinophils near the epithelial surface of the esophagus and occasional findings of several groups of eosinophilic microabscesses and eosinophilic degranulation, the mucous membrane of the esophagus itself is characterized by elongation of the papillae, thickening of the basal layer, expansion of the epithelial intercellular space, and fibrosis of individual mucous membrane layers.^[19–21]^

In this study, using k-means clustering analysis with measurement of RGB values, we confirmed that the differences in the color tone of BCM could be distinguished into “bright” and “dark” BCM. Surprisingly, this result remained consistent when the patients were classified into 2 groups according to their macroscopic characteristics, specifically the change of BCM tone. This raises the question: “why did this difference in the color tone of BCM occur?”

**Figure 4.**
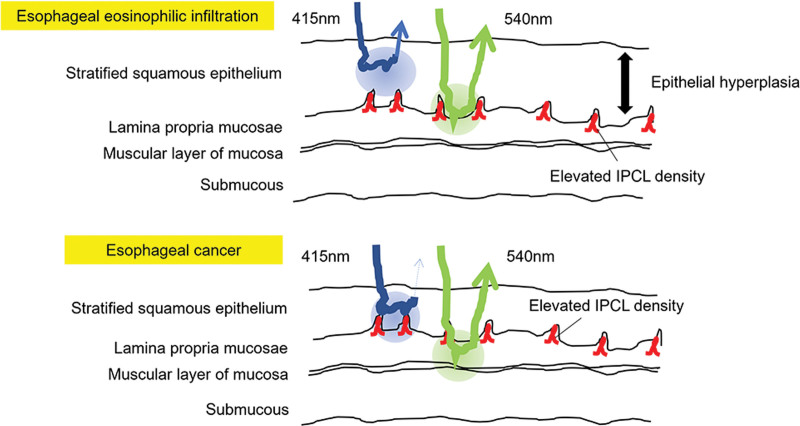
Hypothesis: color changes are influenced by increased intraepithelial papillary capillary loop (IPCL) density and epithelial thickening. Although the 415 nm narrow-band light is well absorbed by IPCL with increased density, the IPCL position is deepened due to epithelial thickening. A short-wavelength light has strong light-scattering properties; therefore, the 415 nm light does not penetrate deeply into the tissues; thus, making it difficult to reach the IPCL. In contrast, the 540 nm light is absorbed lesser by hemoglobin, has a weaker absorption effect, and has a stronger contrast. For bright and dark beige colored mucosa (BCM), bright results are due to the reduced absorption of the 415 nm light caused by epithelial thickening. The absorption of the 415 nm light is lower in esophageal eosinophilic infiltration (EEI) than in esophageal cancer without epithelial thickening; thus, resulting in a stronger reflection. Therefore, EEI is observed as a pale brownish-yellow area rather than the more vivid brownish area as observed in esophageal cancer.

Accordingly, the following hypotheses can be drawn based on the principles of NBI and the histopathology of EEI. The hypothesis is that the increased density of IPCLs and epithelial thickness affected the color change (Fig. 4). Although the 415 nm light is well absorbed by IPCLs with increased density, the IPCLs are pushed deeper due to the thickness of the epithelial layer. Given that a short-wavelength light has strong light-scattering properties, this 415 nm light does not penetrate deeply into the tissues; thus, making it difficult to reach the IPCLs. The 540 nm narrow-band light is absorbed less by Hb, has a weaker absorption effect, and has a stronger contrast than the 415 nm light. As for bright and dark BCM, the results of the bright BCM are supposedly achieved because of the reduced absorption of the 415 nm light caused by the thickened epithelial layer. Additionally, the absorption of the 415 nm light is lower in EEI than in esophageal cancer without epithelial thickening; thus, resulting in a stronger reflection. In other words, EEI is visualized as a pale brownish-yellow color rather than the more vivid brownish area observed in cases of esophageal cancer.

Although we also hypothesized that staining related to red blood cells and Hb from the blood vessels may have some effects, leakage of RBCs was not confirmed in this examination.

We attempted to compare the clinical characteristics, other endoscopic findings, and histology, between bright and dark BCM; however, none of them demonstrated significant difference because our sample size was too small.

Additionally, inspectors performed endoscopy without the knowledge required to recognize EEI and EoE. Furthermore, the endoscopic images were of differing qualities, and each RGB value appears to have been influenced by conditions, such as the brightness of the optical field and the observation angle of the scope. Finally, histopathological evidence could be confirmed in only 1 case in this study.

In conclusion, this study proved that BCM was observed in all cases of EEI and that the difference in the color tone of BCM could be macroscopically distinguished. Additionally, based on our first hypothesis, the difference in the thickness of the epithelial layer might have influenced the tone of BCM (light to dark brown) when the mucosa was viewed using NBI. Furthermore, based on our second hypothesis, there is a possibility that the staining of red blood cells and Hb in blood vessels may have also had some effects.

**Figure 5.**
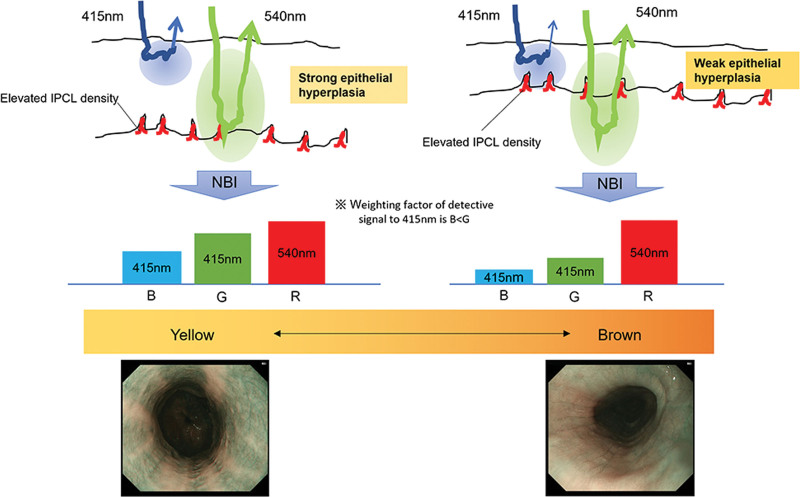
Proposed hypothesis: the tone changes from light to dark brown on narrow-band imaging (NBI) in cases of esophageal eosinophilic infiltration (EEI). The differences in the epithelial thickening may have affected the tone of EEI on NBI.

We propose a new hypothesis regarding the difference in BCM tone based on these observations (Fig. 5). NBI has established evidence, as represented by the vessel plus the surface classification system; however, no further advancements have been made in the classification system based on the color on NBI. To the best of our knowledge, using the tone of BCM on NBI is a potential novel method for diagnosing EoE.

A few limitations of the study should be noted. First, this was a retrospective study. Patients’ information, including symptoms and treatment, was insufficient. This is because patients were not sufficiently interviewed about their symptoms or they did not have adequate laboratory data, although several patients underwent endoscopy for screening. Therefore, sufficient symptom data were lacking for diagnosing patients with EoE. Furthermore, the effectiveness of PPI treatment was not evaluated in some patients, and history of follow-up endoscopy could not be confirmed. As for statistical analyses, it cannot be ruled out that the small sample size due to the single-center retrospective study design may have influenced the study findings.

Although the findings described in this study will contribute to new developments in NBI, further studies are warranted.

## Author contributions

TS performed EGD, detected the new appearance of the esophageal mucosa, conducted the literature search, edited the manuscript, and prepared the figures. YS and YH performed EGD and edited the manuscript. KS prepared the figures and provided an expert pathological opinion. TW edited the manuscript and provided an expert opinion on gastroenterology.

## Acknowledgments

We are grateful to Makoto Igarashi, PhD (OLYMPUS Corporation) for the helpful instructions regarding principles of endoscopy using NBI, and to Professor Masayasu Fukui (Fukuyama Heisei University) for providing the statistical software College Analysis Ver. 7.8.

## Supplementary Material


