# Supplementary figures and images for: Mucosal color changes on narrow-band imaging in esophageal eosinophilic infiltration
Source: Medicine (Baltimore). 2022 Sep 23;101(38):e29891. doi: 10.1097/MD.0000000000029891 (PMC9509114; doi:10.1097/MD.0000000000029891)

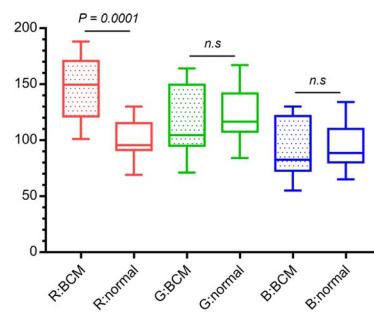

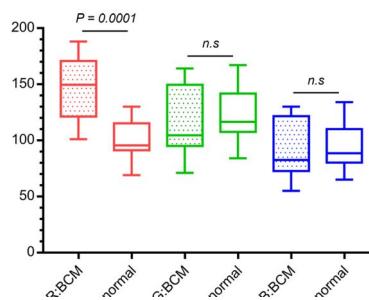

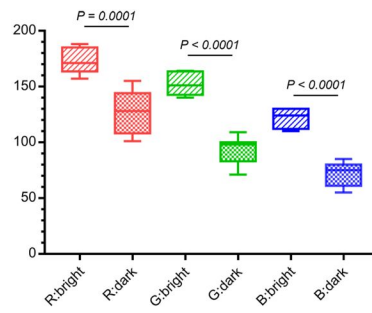

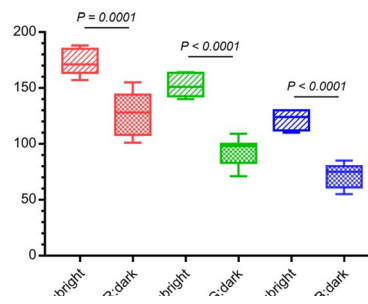

Supplement: Supplementary file 1 [file medi-101-e29891-s001.pdf]
